# Detectability on Plain CT Is an Effective Discriminator between Carcinoma and Benign Disorder for a Polyp >10 mm in the Gallbladder

**DOI:** 10.3390/diagnostics11030388

**Published:** 2021-02-25

**Authors:** Tatsunori Satoh, Masataka Kikuyama, Keiko Sasaki, Hirotoshi Ishiwatari, Shinya Kawaguchi, Junya Sato, Junichi Kaneko, Hiroyuki Matsubayashi

**Affiliations:** 1 Division of Endoscopy, Shizuoka Cancer Center, Shizuoka 411-8777, Japan; h.ishiwatari@scchr.jp (H.I.); ju.sato@scchr.jp (J.S.); j.kaneko@scchr.jp (J.K.); h.matsubayashi@scchr.jp (H.M.); 2 Department of Gastroenterology, Tokyo Metropolitan Cancer and Infectious Diseases Center Komagome Hospital, Tokyo 113-0021, Japan; kikuyama110@yahoo.co.jp; 3 Division of Pathology, Shizuoka Cancer Center, Shizuoka 411-8777, Japan; k.sasaki@scchr.jp; 4 Department of Gastroenterology, Shizuoka General Hospital, Shizuoka 420-8527, Japan; shinya-kawaguchi@i.shizuoka-pho

**Keywords:** gallbladder carcinoma, cholesterol polyp, gallbladder polyp, plain computed tomography

## Abstract

An appropriate diagnosis is required to avoid unnecessary surgery for gallbladder cholesterol polyps (GChPs) and to appropriately treat pedunculated gallbladder carcinomas (GCs). Generally, polyps >10 mm are regarded as surgical candidates. We retrospectively evaluated plain and contrast-enhanced (CE) computed tomography (CT) findings and histopathological features of 11 early GCs and 10 GChPs sized 10–30 mm to differentiate between GC and GChP >10 mm and determine their histopathological background. Patient characteristics, including polyp size, did not significantly differ between groups. All GCs and GChPs were detected on CE-CT; GCs were detected more often than GChPs on plain CT (73% vs. 9%; *p* < 0.01). Sensitivity, specificity, positive and negative predictive values, and diagnostic accuracy for GCs were 73%, 90%, 89%, 75%, and 81%, respectively. On multivariate analysis, lesion detectability on plain CT was independently associated with GCs (odds ratio, 27.1; *p* = 0.044). Histopathologically, GChPs consisted of adipose tissue. Although larger vessel areas in GCs than in GChPs was not significant (52,737 μm^2^ vs. 31,906 μm^2^; *p* = 0.51), cell densities were significantly greater in GCs (0.015/μm^2^ vs. 0.0080/μm^2^; *p* < 0.01). Among GPs larger than 10 mm, plain CT could contribute to differentiating GCs from GChPs.

## 1. Introduction

A gallbladder polyp (GP) is any type of elevated lesion of the gallbladder [1,2]. GPs are classified as benign or malignant according to the results of a histopathological evaluation. Benign GPs include non-tumorous polyps, such as gallbladder cholesterol polyps (GChP) or inflammatory polyps, whereas malignant GPs include gallbladder carcinoma (GC) [3,4]. Among GPs, GChPs are common, while GCs are rare. An appropriate diagnosis is required to avoid unnecessary surgery of GChPs and to provide appropriate treatment for GCs.

To differentiate between benign and malignant GPs, various methods have been reported, including high-resolution abdominal ultrasonography (AUS) [5], contrast-enhanced AUS [6], endoscopic ultrasonography (EUS) [7,8], contrast-enhanced EUS [9], magnetic resonance imaging (MRI) [10], positron emission tomography (PET) [11], and gallbladder bile cytology [12]. Furukawa et al. reported on the difference in the detectability of GCs and GChPs on plain computed tomography (CT) among GPs smaller than 10 mm [13].

The CT number is used to reveal the signal intensity of each part of the image, which is estimated from the difference in the absorption coefficient between water and the object [14,15]. The absorption coefficient is related to the density of the object. If the density of the tissue of the lesion is different, then the CT number is different, and plain CT could be used to differentiate the lesion.

Generally, GPs with a diameter larger than 10 mm are considered to require treatment [16,17,18]. Herein, we evaluated whether detectability on pain CT could become a valid discriminating finding for GCs among GPs >10 mm.

## 2. Materials and Methods

### 2.1. Methods

We retrospectively evaluated the findings on plain CT and contrast-enhanced CT (CE-CT), and the histopathological features of GCs and GChPs. We also compared the detectability on plain CT and the histopathological characteristics associated with their detectability.

### 2.2. Patients

We investigated patients who underwent cholecystectomy at Shizuoka Cancer Center between January 2005 and December 2018. During this period 114 GCs were resected, and all were included in the study. There were 12 early GCs with polypoid growth with a diameter of 10 to 30 mm. One patient without CE-CT due to renal dysfunction was excluded, and 11 out of the 12 early GCs were analyzed in our study. As a control group, 10 consecutive GChPs with a diameter of 10 to 30 mm resected in the same time period were analyzed. The size of the GP was measured on CE-CT. If the patient did not undergo CE-CT or if the GP could not be detected by CE-CT, polyp size was measured on abdominal ultrasound. The study was conducted according to the guidelines of the Declaration of Helsinki, and approved by the Institutional Review Board of Shizuoka Cancer Center (number J2019-17, approved on 23 May 2019).

### 2.3. Evaluation of the CT Scan

**Figure 1:**
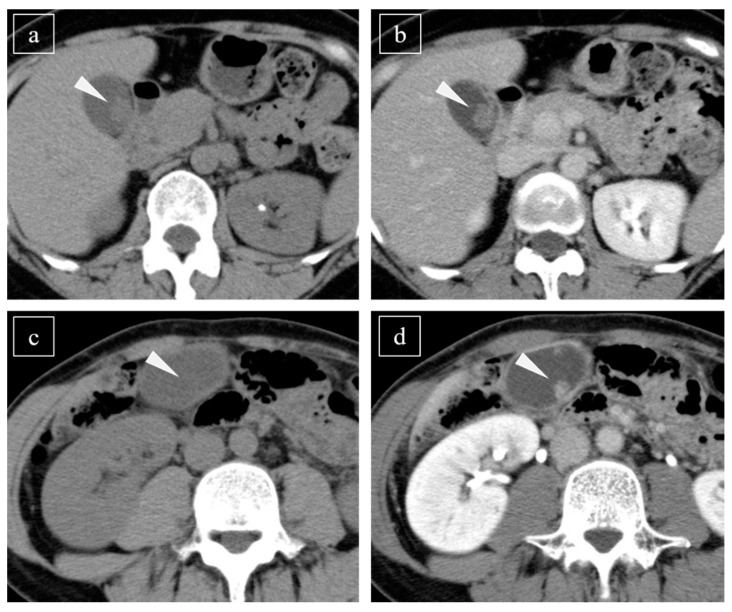
Typical cases of gallbladder polyps detected on plain computed tomography (CT). A pedunculated mass in the gallbladder is recognized using plain CT ((**a**), arrowhead) and contrast-enhanced CT ((**b**), arrowhead); this was determined to be a gallbladder carcinoma. For a gallbladder cholesterol polyp, no lesion is seen in the gallbladder using plain CT ((**c**), arrowhead); however, an enhanced pedunculated mass is detected using contrast-enhanced CT ((**d**), arrowhead).

CT scans were performed using multidetector-row CT with a slice thickness ≤ 5 mm, and the features of CE-CT were evaluated during the late phase (180 s after injection of the contrast medium). All patients except for one GChP patient received CE-CT within 3 months before surgery. Two gastroenterologists (Board Certified Gastroenterologists of The Japanese Society of Gastroenterology and Fellows of the Japanese Society of Internal Medicine) interpreted the detectability of lesions using CT, without information regarding the diagnosis. If both physicians could detect the lesion, the detectability was considered positive (Figure 1). The reproducibility of the interpretation between the two doctors was evaluated using Kappa statistics.

The CT number of each lesion was measured twice, and the mean value was calculated. If the lesion was not detected, then the CT number was measured at the part that was identical to that of the lesion detected on CE-CT. On CE-CT, the CT number of the lesion was measured at the venous phase. Simultaneously, the CT number of bile was measured around the lesion.

### 2.4. Histopathological Assessment

**Figure 2:**
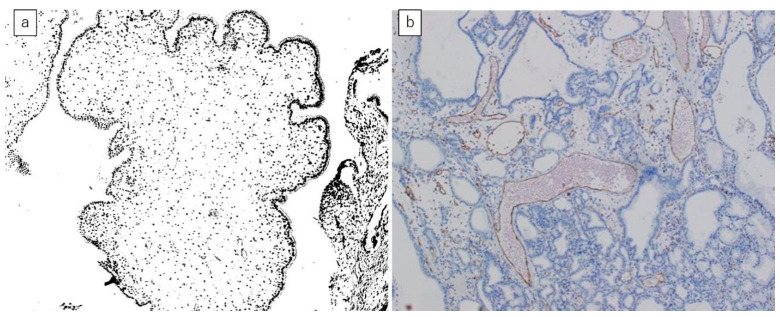
Histopathological examination. (**a**) Representative slide after processing by Image J software (magnification ×10). The nuclease was calculated using Image J software. (**b**) Representative slide after CD31 staining (magnification ×10). The number of vessels and vessel area of each field were measured.

Surgically resected specimens were examined according to the General Rules for Surgical and Pathological Studies on Cancer of the Biliary Tract of the Japanese Society of Biliary Surgery [19]. The number of cells in each field (magnification ×10) was evaluated using Image J software (version 1.52, National Institutes of Health, Bethesda, MD, USA) (Figure 2a). The number and area of vessels in each field (magnification ×10) were also measured. We performed immunochemical staining of historical specimens for CD31, so that we could easily recognize vessels (Figure 2b).

### 2.5. Statistical Analysis

Continuous variables were analyzed using the non-parametric Mann–Whitney U test, and categorical and binary variables were analyzed using Fisher’s exact test due to the relatively small number of cases. All statistical tests were two-tailed and *p* < 0.05 was considered statistically significant. All analyses were performed using R version 3.4.1 (R Foundation, Vienna, Austria).

## 3. Results

### 3.1. Patient Characteristics

Age, sex, polyp size, carcinoembryonic antigen value, and CA19-9 value did not significantly differ between the GC and GChP groups (Table 1). There were also no significant differences in the indication for workup and medical history between the two groups (Table 1).

### 3.2. Detectability of Lesions Using Plain CT and CE-CT

All GCs and GChPs could be detected on CE-CT, whereas GCs were detected significantly more often than GChPs on plain CT (73% vs. 10%; *p* < 0.01) (Table 2). Three cases of GC (3/11; 27.3%) were not recognized on plain CT. The difference between the CT number of the lesion and bile within the gallbladder is more than 5 in all the detected GPs. The sensitivity, specificity, positive predictive value, negative predictive value, and diagnostic accuracy for GCs were 73%, 90%, 89%, 75%, and 81%, respectively. The detectability of each individual reader for GPs was correctly matched (κ = 1).

### 3.3. Multivariate Analysis

Detectability on plain CT was the only independent factor for GC (odds ratio, 27.1; *p* = 0.045) (Table 3).

### 3.4. Histopathological Evaluation

GChPs consisted of adipose tissue covered by monolayer epithelium. In contrast, GCs were formed by papillary or tubular proliferation of tumorous epithelium with thin connective tissue. Cell densities in the lesions including the mucosal layer were significantly greater in GCs than in GChPs (0.015/μm^2^ vs. 0.0080/μm^2^; *p* < 0.01) (Table 4). Although the vessel areas of each tissue field (magnification ×10) were larger for GCs than for GChPs (52,737 μm^2^ vs. 31,906 μm^2^; *p* = 0.51), the difference did not reach significance (Table 4). Cell densities were also significantly greater in detected GPs than in undetected GPs (0.015/μm^2^ vs. 0.0083/μm^2^; *p* < 0.01) (Table 5). The cell density was significantly associated with the plain CT value of GPs (ρ = 0.45, *p* = 0.04) (Table 6) and the detectability on plain CT.

## 4. Discussion

### 4.1. Need for Differentiation between GC and Noncancerous GP

The Clinical Practice Guideline for the Management of Biliary Tract Cancers [20] indicates that the best treatment for early GC is surgery. Open surgery is recommended if GC is strongly suspected, even if the tumor is classified as pT1, because laparoscopic surgery could induce port site recurrence or intraperitoneal dissemination [21,22,23]. Moreover, a pT1 tumor could involve lymph node metastasis to the hepatoduodenal lymph node; surgery with lymphadenectomy is therefore recommended [24,25]. However, surgery is not required for patients with a GChP or other types of noncancerous polyps [2], and even lowly invasive laparoscopic surgery is unnecessary for patients with GChPs; therefore, it is necessary to appropriately diagnose GPs.

### 4.2. Methods of the Differential Diagnosis for GPs

Many reports have described that a GP larger than 10 mm could be a GC [16,17,18], and the Clinical Practice Guideline for the Management of Biliary Tract Cancers recommends surgical treatment for such lesions [20]. However, GPs larger than 10 mm are not always cancer, which is why an appropriate diagnosis is important.

Various reports have indicated the differential diagnosis of GPs [6,9,11,12,13,26,27]. Differential diagnosis using detectability on plain CT has several advantages. First, the identification of GPs on plain CT is simple, and this method could easily differentiate malignant from benign in gallbladder polyps compared to other imaging methods such as CE-CT or US. Although this study has a small sample size, the detectability of each individual reader was matched. Second, plain CT is easier and less invasive than endoscopic procedures such as CE-CT, ERCP, or EUS.

### 4.3. Correlation between Histopathological Results and CT Findings

The number and area of the nuclei were higher in GCs than in GChPs. This suggests that the cell densities are greater for GCs than those for GChPs, thereby contributing to the larger CT number for GCs. The CT number is calculated by the difference in the absorption coefficient of the X-ray between water and tissue; therefore, the density of the tissue is related to the CT number, and higher-density tissue has a higher CT number [14].

Moreover, GChPs have abundant lipids in the mucosa propria, and the lipid content generally lowers the CT number of the tissue. These things are associated with the result that GChP could be rarely recognized.

The vessel areas of each tissue field were larger for GCs than for GChPs. The CT attenuation of blood reflects the concentration proteins including hemoglobin which has a high density. However, the difference between GCs and GChPs was not statistically significant.

### 4.4. A Clinical Strategy for GPs

**Figure 3:**
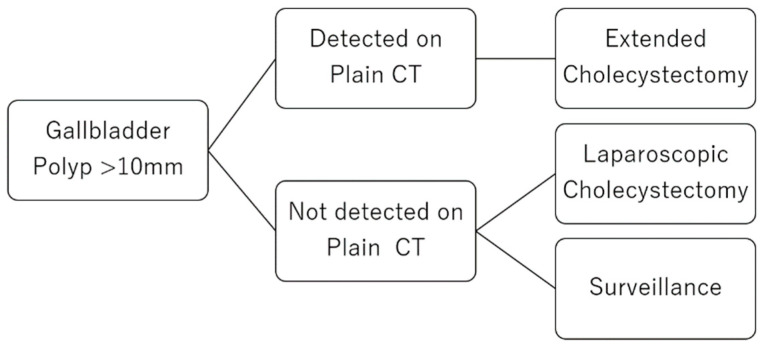
Proposal of a clinical strategy for diagnosing gallbladder polyps (GPs). If a GP is larger than 10 mm is recognized on plain CT, GC is suspected, and open surgery is recommended. A GP not detected on plain CT could be considered a GChP, which should be observed regularly. Additionally, laparoscopic surgery may be recommended.

If a GP larger than 10 mm is recognized on plain CT, then GC is suspected, and open surgery is recommended (Figure 3). A GP not detected on plain CT could be considered a GChP and should be observed regularly, because in our study, three cases of epithelial GC (3/11; 27.3%) were not recognized on plain CT. It is important to remember that plain CT findings are not always reliable.

### 4.5. Limitations

Our study was limited by its retrospective, single-center design. Additionally, it was limited by the small number of cases. A further prospective confirmatory study is necessary to clarify our findings.

## 5. Conclusions

Among GPs larger than 10 mm, plain CT could contribute to differentiating GCs from GChPs, i.e., GCs were detected significantly more often on plain CT than were GChPs, as GChPs were scarcely recognized. The ability to differentiate between the two depended on the lipid content and cell density of the lesion.

## Figures and Tables

**Table 1:** Characteristics of patients with early gallbladder carcinoma and gallbladder cholesterol polyps.

	GC (*n* = 11)	GChP (*n* = 10)	*p* Value
Age, years	63 (40–90)	60.5 (45–76)	0.78
Male, number	3 (27)	3 (30)	1.0
Indication for workup			
Symptoms	3 (27)	1 (10)	0.59
Incidental	8 (72)	9 (90)	0.59
Medical checkup/screening	5 (45)	4 (40)	
Initial examination for other disease	1 (9)	3 (30)	
Surveillance for other disease	2 (18)	2 (20)	
History of follow up	4 (36)	6 (60)	0.40
Tumor size, mm	18 (10–28)	14.5 (10–20)	0.23
CEA, ng/mL	3.0 (0.9–17)	2.2 (0.8–49)	0.65
CA19-9, U/mL	15 (2–58)	11 (2–45)	0.50

Numbers are shown as *n* (%) or median (range). CEA, carcinoembryonic antigen; GC, gallbladder carcinoma; GChP, gallbladder cholesterol polyp.

**Table 2:** Lesion detectability on plain CT and CE-CT.

	GC (*n* = 11)	GChP (*n* = 10)	*p* Value
Detected on plain CT	8 (73)	1 (10)	<0.01
Detected on CE-CT	11 (100)	10 (100)	1.0
CT value of the lesions on plain CT	28.5 (10–37.5)	12 (4.5–23)	<0.01
CT value of the lesions on CE-CT	80 (40.5–116)	67.25 (32–106)	0.35
CT value of bile around the lesion	11 (7–20.5)	12 (8.5–26)	0.34

Numbers are shown as *n* (%) or median (range). CT, computed tomography; CE-CT, contrast-enhanced enhanced computed tomography; GC, gallbladder carcinoma; GChP, gallbladder cholesterol polyp.

**Table 3:** Univariate and multivariate analyses for gallbladder carcinoma.

				Univariate Analysis			Multivariate Analysis		
Variable	Cutoff	GC	GChP	OR	95% CI	*p* Value	OR	95% CI	*p* Value
Age (years)	>63	3	5	1.94	0.32–11.8	0.47	0.58	0.035–9.52	0.70
	≤63	7	5	1			1		
CEA (ng/mL)	>2.9	6	3	2.80	0.46–16.9	0.26	1.97	0.10–37.4	0.65
	≤2.9	5	7	1			1		
CA19-9 (U/mL)	>10	8	5	2.67	0.43–16.4	0.29	1.27	0.089–18.2	0.86
	≤10	3	5	1			1		
Detected on PCT	Yes	8	1	24.0	2.06–280	0.011	27.1	1.07–685	0.045
	No	3	9	1			1		
CT value on CE-CT	>68	8	5	2.67	0.43–16.4	0.29	0.86	0.034–21.8	0.93
	≤68	3	5	1			1		

Numbers are shown as *n* (%) or median (range). CEA, carcinoembryonic antigen; CI, confidence interval; CT, computed tomography; PCT, plain CT; CE-CT, contrast-enhanced CT; GC, gallbladder carcinoma; GChP, gallbladder cholesterol polyp; OR, odds ratio.

**Table 4:** Histopathological assessment of vessels and cell densities between gallbladder carcinoma and gallbladder cholesterol polyp.

	GC	GChP	*p* Value
Vessel area *, μm^2^	52,737 (4292–635,267)	31,906 (9594–370,033)	0.51
Number of vessels *	24 (9–32)	13.5 (7–35)	0.72
Cell densities *, *n*/μm^2^	0.015 (0.0084–0.024)	0.0080 (0.0050–0.018)	<0.01

Numbers are shown as median (range). GC, gallbladder carcinoma; GChP, gallbladder cholesterol polyp. * Per field using magnification ×10.

**Table 5:** Histopathological assessment of vessels and cell densities between detected and undetected gallbladder polyp.

	Detected GPs	Undetected GPs	*p* Value
Vessel area *, μm^2^	52,165 (13955–635,267)	48,053 (4292–370,033)	0.86
Number of vessels *	24 (9–32)	13.5 (7–39)	0.52
Cell densities *, *n*/μm^2^	0.015 (0.0085–0.024)	0.0083 (0.0050–0.018)	<0.01

Numbers are shown as median (range). GPs, gallbladder polyps. * Per field using magnification ×10.

**Table 6:** Correlation between histopathologic measures and plain CT values.

	ρ	*p* Value
Vessel area	−0.029	0.90
Number of vessels	−0.053	0.82
Cell densities	0.45	0.04

The association between histopathologic measures and the plain CT values of gallbladder polyps was evaluated using Spearman’s rank correlation coefficient.

## Data Availability

The data presented in this study are available on request from the corresponding author. The data are not publicly available due to privacy concerns.
